# A trans‐omic Mendelian randomization study of parental lifespan uncovers novel aging biology and therapeutic candidates for chronic diseases

**DOI:** 10.1111/acel.13497

**Published:** 2021-10-27

**Authors:** Nicolas Perrot, William Pelletier, Jérôme Bourgault, Christian Couture, Zhonglin Li, Patricia L. Mitchell, Nooshin Ghodsian, Yohan Bossé, Sébastien Thériault, Patrick Mathieu, Benoit J. Arsenault

**Affiliations:** ^1^ Centre de recherche de l’Institut universitaire de cardiologie et de pneumologie de Québec Québec QC Canada; ^2^ Department of Medicine Faculty of Medicine Université Laval Québec QC Canada; ^3^ Department of Molecular Medicine Faculty of Medicine Université Laval Québec QC Canada; ^4^ Department of Molecular Biology, Medical Biochemistry and Pathology Faculty of Medicine Université Laval Québec QC Canada; ^5^ Department of Surgery Faculty of Medicine Université Laval Québec QC Canada

**Keywords:** Mendelian Randomization, Metabolomics, Parental Lifespan, Proteomics, Transcriptomics

## Abstract

The study of parental lifespan has emerged as an innovative tool to advance aging biology and our understanding of the genetic architecture of human longevity and aging‐associated diseases. Here, we leveraged summary statistics of a genome‐wide association study including over one million parental lifespans to identify genetically regulated genes from the Genotype‐Tissue Expression project. Through a combination of multi‐tissue transcriptome‐wide association analyses and genetic colocalization, we identified novel genes that may be associated with parental lifespan. Mendelian randomization (MR) analyses also identified circulating proteins and metabolites causally associated with parental lifespan and chronic diseases offering new drug repositioning opportunities such as those targeting apolipoprotein‐B‐containing lipoproteins. Liver expression of *HP*, the gene encoding haptoglobin, and plasma haptoglobin levels were causally linked with parental lifespan. Phenome‐wide MR analyses were used to map genetically regulated genes, proteins and metabolites with other human traits as well as the disease‐related phenome in the FinnGen cohorts (*n* = 135,638). Altogether, this study identified new candidate genes, circulating proteins and metabolites that may influence human aging as well as potential therapeutic targets for chronic diseases that warrant further investigation.

## Graphical abstract

Schematic overview of the analytical framework used to identify novel biological determinants of lifespan and potential drug targets for aging‐associated diseases using genome‐wide association study summary statistics of >1 million parental lifespans.

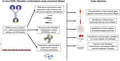

## 1 INTRODUCTION

Increasing lifespan and promoting healthy living into old age are among top priorities of health care systems around the world. Over the past decades, several policies have been set in place to improve sanitation, access to care and control risk factors for premature mortality. In Western societies, however, although lifespan is steadily improving, an increasing proportion of the population is affected by chronic diseases and multimorbidity, which may be attributable to a significant extent to poor lifestyles as well as environmental and socio‐economic factors. Unprecedented efforts will be needed to target these risk factors and decrease age‐associated disease burden and improve the quality of life of aging populations. Drugs aimed at improving lifespan may represent additional options that could be used to prevent aging‐associated diseases. Increasing evidence suggests that drug targets with genetic support are more likely to be efficient and to be approved by regulatory authorities (Nelson et al., 2015). A better characterization of longevity genomics could therefore contribute to the identification of drug development strategies to delay the onset of aging‐associated diseases.

The genetics revolution offers new opportunities to foster our understanding of complex traits such as human longevity (Cotto et al., 2018; Melzer et al., 2020). The definition of longevity in human genetic studies is highly debated and the lack of a universally recognized definition increases the possibility of biases and limits external validation. Studying the genetic architecture of long‐lived individuals has shed some light on the loci at which genetic variation may influence human longevity. However, the most comprehensive meta‐analysis of genome‐wide association studies (GWAS) of survival percentiles only reported one locus (*APOE*) to be robustly associated with human longevity (Deelen et al., 2019). On the other hand, other longevity traits such as parental lifespan (the lifespan of deceased or long‐lived parents) have also been used to uncover longevity genes. In the most comprehensive GWAS of parental lifespan to date, Timmers et al. (2019) reported several new loci associated with parental lifespan, including *APOE*. Although it has been suggested that human longevity is not simply limited to the absence of life‐threatening diseases, many of the reported genetic variants associated with lifespan are also associated with chronic diseases.

Mendelian randomization (MR) is a burgeoning field of research that draws on the use of genetic variants as instruments to assess potentially causal relationships between a wide variety of exposures such as risk factors or drug targets and outcomes such as age‐related disease or human longevity (Hemani et al., 2018). By taking advantage of the naturally randomized allocation of genetic variation, MR is not subject to many of the biases of observational studies such as reverse causality or random measurement error and less susceptible to confounding and reverse causality. The recent public release of a wide variety of deeply phenotyped gene‐trait association datasets has enabled the use of multi‐omic datasets as exposures in MR studies. However, few studies have investigated the long‐term consequences of lifelong exposure to over‐ or under‐expressed genes (eGenes), plasma proteins, lipoproteins or metabolites on human lifespan and associated age‐related chronic disease. In this study, we aimed at discovering and harnessing novel biological determinants of human longevity by identifying eGenes through a transcriptome‐wide association study (TWAS) as well as genetically regulated circulating proteins (eProteins) and metabolites (eMetabolites) using a highly translational MR framework. We performed a TWAS for parental lifespan leveraging expression Quantitative Trait Loci (eQTL) data from 43 non‐sex‐specific tissues to identify eGenes associated with parental lifespans. In light of recent investigations reporting that the majority of eQTLs do not necessarily influence protein levels (He et al., 2020; Yang et al., 2020) we used MR to identify key eProteins, and eMetabolites, that may influence human lifespan. In order to discover the role of these genetically regulated traits in human homeostasis while at the same time determining whether they could be effectively and safely targeted to promote healthy aging and human longevity, we report MR findings across the human disease‐related phenome.

## 2 RESULTS

### 2.1 Identification of eGenes associated with parental lifespan

**FIGURE 1:**
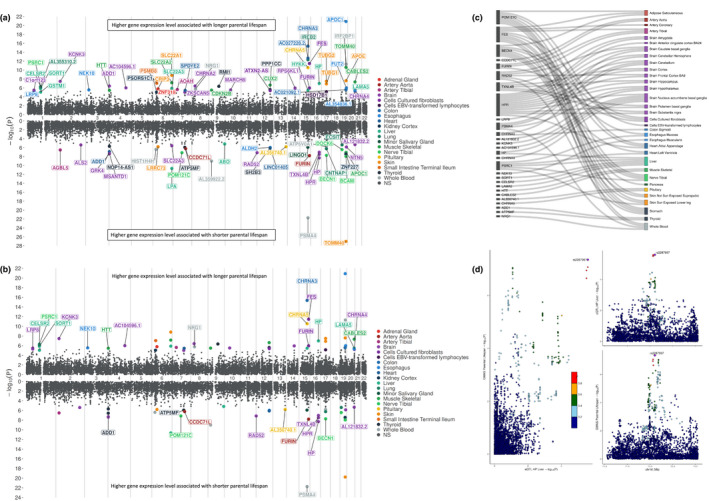
A multi‐tissue transcriptome‐wide association study of parental lifespan. (a) Miami plot depicting the results of transcriptome‐wide association studies of parental lifespan in multiple tissues before filtering out eGenes without evidence of genetic colocalization. Each dot represents the effect of an eGene on parental lifespan and the top tissue underlying the signal is shown. eGenes negatively associated with parental lifespan are above the baseline and eGenes positively associated with parental lifespan are below the baseline. Some tissues (for instance those in the brain) were pooled in the legend to facilitate visualization of the tissues responsible for the eGene‐parental lifespan associations. (b) Miami plot depicting the results of transcriptome‐wide association studies of parental lifespan in multiple tissues after filtering out eGenes without evidence of genetic colocalization (posterior probability of statistical colocalization <0.75) and after excluding genes found in pleiotropic regions such as *HLA*, *ABO* and *APOE*. (c) Sankey diagram depicting tissues that are responsible for the eGene‐trait associations. This analysis is based on 43 non‐sex‐specific tissues from GTEx. (d) LocusCompare plot depicting colocalization of the top SNP associated with liver *HP* expression and parental lifespan. Each dot represents a single‐nucleotide polymorphism (SNP) at the HP locus. In the left panel, these SNPs are plotted to represent their effect on HP expression (top right) against their effect on parental lifespan (bottom right)

We performed a TWAS using the MetaXcan framework (Barbeira et al., 2018) to identify potentially causal effects of eGenes on parental lifespan using GWAS summary‐level statistics of the UK Biobank and LifeGen consortium meta‐analysis (methods) (Timmers et al., 2019). TWAS is a type of 2‐sample MR study that takes advantage of all cis‐acting single‐nucleotide polymorphisms (SNPs). These SNPs are often in linkage disequilibrium (LD) with each other and using all SNPs for TWAS can ensure optimal statistical power, in contrast to classical MR studies that use one or multiple independent SNPs. This analytical approach uses eQTL mapping from the Genotype‐Tissue Expression (GTEx) project across 43 non‐sex‐specific tissues to estimate gene‐level association with summary‐level GWAS results. This strategy led to the identification of new potential eGenes for parental lifespan after correction for multiple testing (Figure 1a). A detailed description of the association of eGenes‐parental lifespan associations across all tissues is presented in Table S1. Because TWAS prioritizes multiple genes, some eGenes could be non‐causal, owing to sharing of eQTLs or co‐regulated genes. TWAS may thus be prone to false positives and spurious gene prioritization, especially in the absence of genetic colocalization, that is, when the lead genetic variant driving gene expression is not among the lead GWAS variants. We therefore took additional steps using genetic colocalization to control for spurious prioritization. After excluding genes with a posterior probability of statistical colocalization PPH4 < 0.75 and after excluding genes found in pleiotropic regions such as *HLA*, *ABO* and *APOE*, the number of parental lifespan eGenes was reduced to 30, spanning 17 loci (Figure 1b). Details on strength of the association, colocalization of these eGenes with parental lifespan as well as the lead tissue (i.e., tissue providing the strongest eGenes‐parental lifespan estimate from MetaXcan) are provided in Table 1. Posterior probabilities of genetic colocalization (PPH4) using a wider range of priors are presented in Table S2. We identified tissue‐specific expression regulation of several known parental lifespan genes and revealed potentially new parental lifespan genetic signals at the *LRP8* (ApoER2), *NEK10*, *CCDC71L*, *NRG1* and *RAD52* loci. Although the use of statistical colocalization helped prioritize several genes, the causal gene potentially linked with parental lifespan could not be identified in all genetic regions. This is the case for the *CELSR*‐*PSRC1*‐*SORT1*, *FURIN*‐*FES*, *TXNL4B*‐*HP*‐*HPR* and *LAMA5*‐*AL121832*.*2*‐*CABLES2*‐*CHRNA4* regions, within which the lead parental lifespan variant was linked with the expression of two or more genes. Multivariable MR, a MR technique used to identify the causal exposure accounting for potential confounders, did not identify the causal eGene from these loci (data not shown). We also observed that among colocalized eGenes for parental lifespan FURIN and HP, gene expression in one tissue was positively linked with parental lifespan while negatively linked with parental lifespan in another tissue. A Sankey diagram presents tissues underlying the genetic signals of colocalized eGenes (Figure 1c). Table S3 presents the results of a classical MR approach that reports associations between the level of expression of each eGene presented in Table 1 (in the tissue identified in Table 1) and parental lifespan using inverse variance weighted (IVW)‐MR and other outlier robust MR methods. This analysis revealed that most of the colocalized eGenes were nominally associated with parental lifespan with the exception of *LRP8* and *BECN1*. We also performed similar analyses using data on whole blood gene expression levels from 31,684 individuals of the eQTLGen consortium as exposure (Võsa et al., 2018). Although only 17 of the 28 independent and colocalized eGenes could be evaluated using MR, we found that most eGenes showed nominal associations with parental lifespan with the exception of *LRP8*, *POM121C*, *FURIN*, *TXNL4B* and *LAMA5* (Table S4). These analyses revealed the important of considering tissue specificity and TWAS methods over classical MR for the identification of novel eGene‐trait associations. In order to gain insight into potential tissue specificity of the parental lifespan associated eGenes, we obtained the tissue‐specific gene expression metric (τ) as described by Kryuchkova‐Mostacci and Robinson‐Rechavi (2017). This analysis revealed that several of the eGenes had tissue‐specific expression (τ ≥0.80), including the *HP* gene, which appears to be liver‐specific, in accordance to our initial TWAS finding (Table S5). Other tissue‐specific eGenes include *KCNK3*, *NRG1*, *NEK10*, *CHRNA3*/*5* and *CHRNA4*. We used LocusCompare (Liu et al., 2019) to depict colocalization events within our framework and present as an example the colocalization of the top SNP associated with liver *HP* expression and parental lifespan (Figure 1d).

**TABLE 1:** Significant eGene‐parental lifespan associations from a transcriptome‐wide association study of parental lifespan after filtering out eGenes without evidence of genetic colocalization

Gene	Ensembl ID	Chromosome band	Lead tissue	TWAS Z‐score	*p*‐value	*p*‐value threshold	COLOC PP4
LRP8	ENSG00000157193.15	1p32	Brain Caudate basal ganglia	4.566	4.97e−06	7.67e−06	0.761
CELSR2	ENSG00000143126.7	1p13	Liver	5.033	4.83e−07	9.19e−06	0.987
PSRC1	ENSG00000134222.16	1p13	Nerve Tibial	5.032	4.86e−07	3.66e−06	0.986
SORT1	ENSG00000134243.11	1p13	Liver	4.835	1.33e−06	9.19e−06	0.987
KCNK3	ENSG00000171303.6	2p23	Brain Nucleus accumbens basal ganglia	5.519	3.41e−08	7.11e−06	0.950
NEK10	ENSG00000163491.16	3p24	Esophagus Muscularis	4.685	2.80e−06	4.39e−06	0.768
ADD1	ENSG00000087274.16	4p16	Thyroid	−5.141	2.74e−07	3.68e−06	0.825
HTT	ENSG00000197386.10	4p16	Muscle Skeletal	4.642	3.45e−06	4.53e−06	0.926
AC104596.1	ENSG00000250326.1	4q31	Brain Nucleus accumbens basal ganglia	4.894	9.87e−07	7.11e−06	0.815
POM121C	ENSG00000272391.5	7q11	Nerve Tibial	−5.379	7.48e−08	3.66e−06	0.996
ATP5MF	ENSG00000241468.7	7q22	Thyroid	−4.854	1.21e−06	3.68e−06	0.928
CCDC71L	ENSG00000253276.2	7q22	Artery Aorta	−4.994	5.90e−07	5.30e−06	0.975
NRG1	ENSG00000157168.18	8p12	Whole Blood	4.781	1.74e−06	4.94e−06	0.850
RAD52	ENSG00000002016.17	12p13	Brain Caudate basal ganglia	−5.383	7.34e−08	7.67e−06	0.976
AL356740.1	ENSG00000267868.1	13q34	Pituitary	−4.796	1.62e−06	5.97e−06	0.752
PSMA4	ENSG00000041357.15	15q25	Whole Blood	−9.749	1.86e−22	4.94e−06	0.948
CHRNA5	ENSG00000169684.13	15q25	Pituitary	6.650	2.93e−11	5.97e−06	0.889
CHRNA3	ENSG00000080644.15	15q25	Colon Sigmoid	8.129	4.33e−16	5.71e−06	0.815
FURIN	ENSG00000140564.11	15q26	Artery Aorta	−5.650	1.61e−08	5.30e−06	0.896
FURIN	ENSG00000140564.11	15q26	Cells Cultured fibroblasts	4.882	1.05e−06	4.16e−06	0.785
FES	ENSG00000182511.11	15q26	Cells Cultured fibroblasts	6.959	3.41e−12	4.16e−06	0.991
TXNL4B	ENSG00000140830.8	16q22	Brain Putamen basal ganglia	−5.335	9.57e−08	7.65e−06	0.890
HP	ENSG00000257017.8	16q22	Brain Nucleus accumbens basal ganglia	−5.844	5.10e−09	7.11e−06	0.943
HP	ENSG00000257017.8	16q22	Liver	5.324	1.02e−07	9.19e−06	0.849
HPR	ENSG00000261701.6	16q22	Brain Cerebellum	−5.531	3.19e−08	5.56e−06	0.991
BECN1	ENSG00000126581.12	17q21	Nerve Tibial	−5.624	1.87e−08	3.66e−06	0.925
LAMA5	ENSG00000130702.15	20q13	Liver	4.657	3.21e−06	9.19e−06	0.832
AL121832.2	ENSG00000273619.1	20q13	Brain Hypothalamus	−4.572	4.82e−06	9.16e−06	0.871
CABLES2	ENSG00000149679.11	20q13	Muscle Skeletal	5.468	4.56e−08	4.53e−06	0.967
CHRNA4	ENSG00000101204.16	20q13	Brain Nucleus accumbens basal ganglia	4.528	5.95e−06	7.11e−06	0.920

### 2.2 Identification of eProteins associated with parental lifespan

**FIGURE 2:**
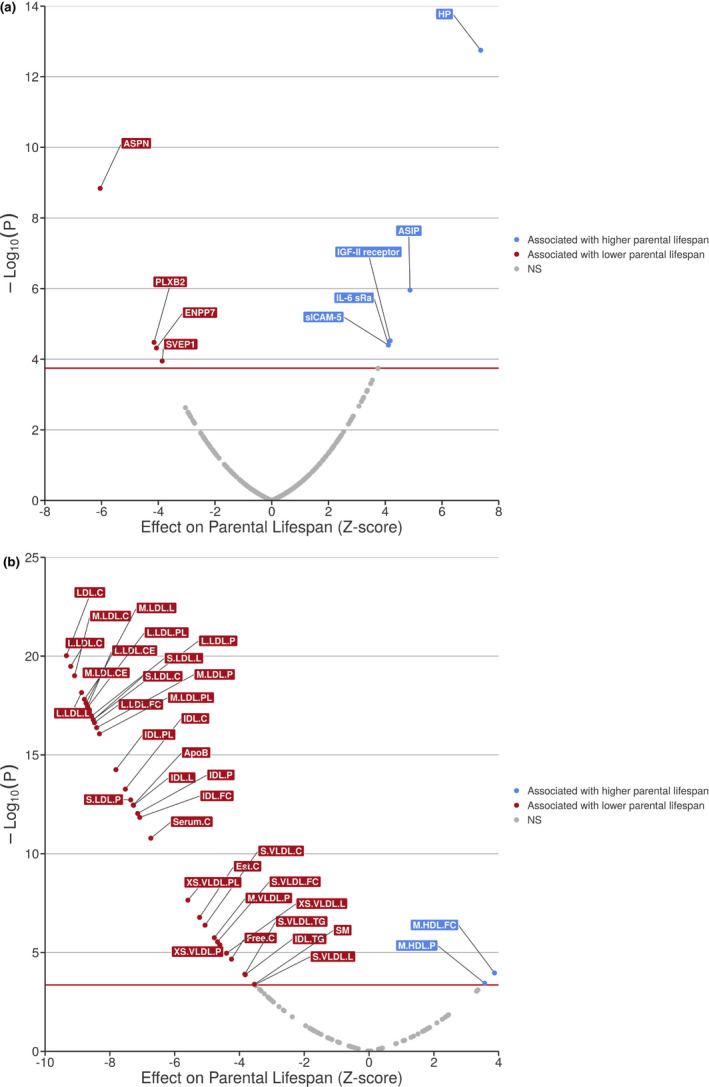
Proteome‐ and metabolome‐wide Mendelian randomization analysis of parental lifespan. Volcano plots representing the association between plasma eProteins from the study of Sun et al. (a) and eMetabolites from Kettunen et al. (b) and parental lifespan using inverse variance weighted Mendelian randomization. eProteins and eMetabolites in blue represent those positively associated with parental lifespan and those in red are negatively associated with parental lifespan

Proteins are the target of most medicines. In order to identify circulating factors causally linked with parental lifespan, simultaneously representing therapeutic targets for aging‐associated diseases, we performed a systematic screen of the human plasma proteome of the INTERVAL cohort (Sun et al., 2018) using MR. We first obtained robust instruments for protein levels by identifying proteins with ≥4 cis‐acting independent variants (*r* ^2^ < 0.1) strongly associated with protein levels (*p*‐value <5e^−8^). A total of 279 circulating proteins were available for MR analyses using these criteria. We then used IVW‐MR to determine the association between these circulating proteins and parental lifespan. Nine proteins emerged as causal mediators of parental lifespan in the proteome‐wide MR analysis, including haptoglobin (*HP* gene on chromosome 16q22), which has also been identified by GWAS and TWAS of liver‐parental lifespan associations (Figure 2a). Other proteins identified as causal mediators of parental lifespan include asporin (*ASPN* gene on chromosome 9q22), agouti‐signaling protein (*ASIP* gene on chromosome 20q11), the soluble receptor of insulin‐like growth factor 2 (*IGF2R* gene on chromosome 6q25), plexin‐B2 (*PLXNB2* gene on chromosome 22q13), interleukin‐6 receptor subunit alpha (*IL6R* gene on chromosome 1q21), soluble intercellular adhesion molecule‐5 (*ICAM5* gene on chromosome 19p13), ectonucleotidase phosphodiesterase 7 (*ENPP7* gene on chromosome 7q25) and Sushi, von Willebrand factor type A, EGF and pentraxin domain‐containing protein 1 (*SVEP1* gene on chromosome 9q31). In order to determine whether these associations were robust to pleiotropy and outliers, we used additional outlier robust (MR‐PRESSO) and modeling MR methods (MR‐Egger and contamination mixture). The association of these proteins with parental lifespan using these methods are presented in Table S6. Results of this analysis suggest that the causal association of these proteins with parental lifespan is robust to outliers. The MR method however detected evidence of horizontal pleiotropy for one protein, IL6‐sRA. In order to determine if there was general agreement between eProteins and eGenes, we investigated whether the top pQTLs for eProteins also influenced gene expression levels in the GTEx tissues. Results presented in Table S7 suggest that most pQTLs were indeed eQTLs, but in a tissue dependent manner. Altogether, this analysis identified five circulating “protective” proteins that may be positively associated with parental lifespan and four circulating proteins that may be negatively associated with lifespan.

### 2.3 Identification of eMetabolites associated with parental lifespan

We applied a similar MR framework as above to identify eMetabolites including lipoprotein metabolomic parameters that could be causally associated with parental lifespan using a GWAS‐metabolomics dataset, previously described by Kettunen et al. (2016). The association between 123 metabolites from this datasets and parental lifespan was investigated using IVW‐MR (Figure 2b). After correction for multiple testing, eMetabolites associated with parental lifespan included several parameters linked to very‐low‐density lipoprotein (VLDL) and low‐density lipoprotein (LDL) physicochemical properties (e.g., apolipoprotein B, cholesterol content and total VLDL/LDL particle number), sphingomyelin and the number of high‐density lipoprotein particles of medium size (M:HDL‐P). The same MR methods that enabled us to identify causal proteins associated with parental lifespan were used and no evidence of horizontal pleiotropy or distortion of the causal estimates was detected using these methods (Table S8). Overall, results of this analysis suggest that elevated apolipoprotein‐B‐containing lipoprotein levels are strongly associated with shorter parental lifespan.

### 2.4 Investigation of genetic colocalization across multiple traits at the HP locus

**FIGURE 3:**
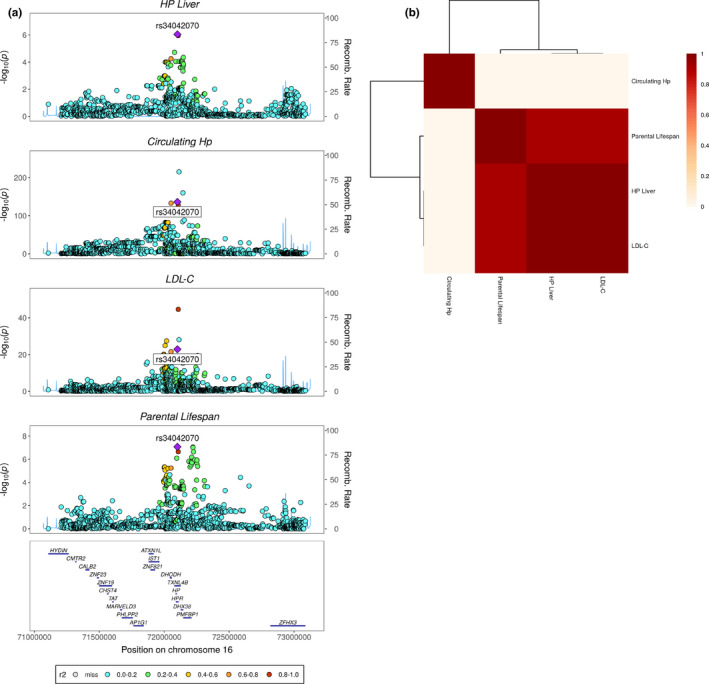
Statistical colocalization at the *HP* locus. (a) Regional association plot highlighting the lead SNP associated with liver *HP* expression (rs34042070) and circulating haptoglobin levels, low‐density lipoprotein cholesterol levels and parental lifespan with color key indicating *r* ^2^ with the lead SNP. (b) Heatmap depicting the posterior probabilities of colocalization between each trait pair

Given that the only parental lifespan signal supported by GWAS, TWAS and proteome‐wide MR was at *HP*, the gene encoding haptoglobin (Hp), and that variation at the *HP* locus was linked with hyperlipidemia, we investigated genetic colocalization across multiple traits at the *HP* locus. For this purpose, we used a Bayesian algorithm called Hypothesis Prioritization in multi‐trait Colocalization (HyPrCOLOC) (Foley et al., , 2021). In order to reduce the risk associated with spurious association from overlapping dataset, we used GWAS summary statistics from the Global Lipids Genetics Consortium for LDL‐C. Results presented in Figure 3a suggest strong evidence of genetic colocalization between liver *HP* expression, circulating LDL levels, and parental lifespan. Upon further investigation, we found that liver *HP* expression, circulating LDL levels, parental lifespan but not circulating Hp levels showed evidence of genetic colocalization with a posterior probability of colocalization of 0.96 (Figure 3b).

### 2.5 Identification of drug repositioning opportunities for human lifespan and chronic diseases

**FIGURE 4:**
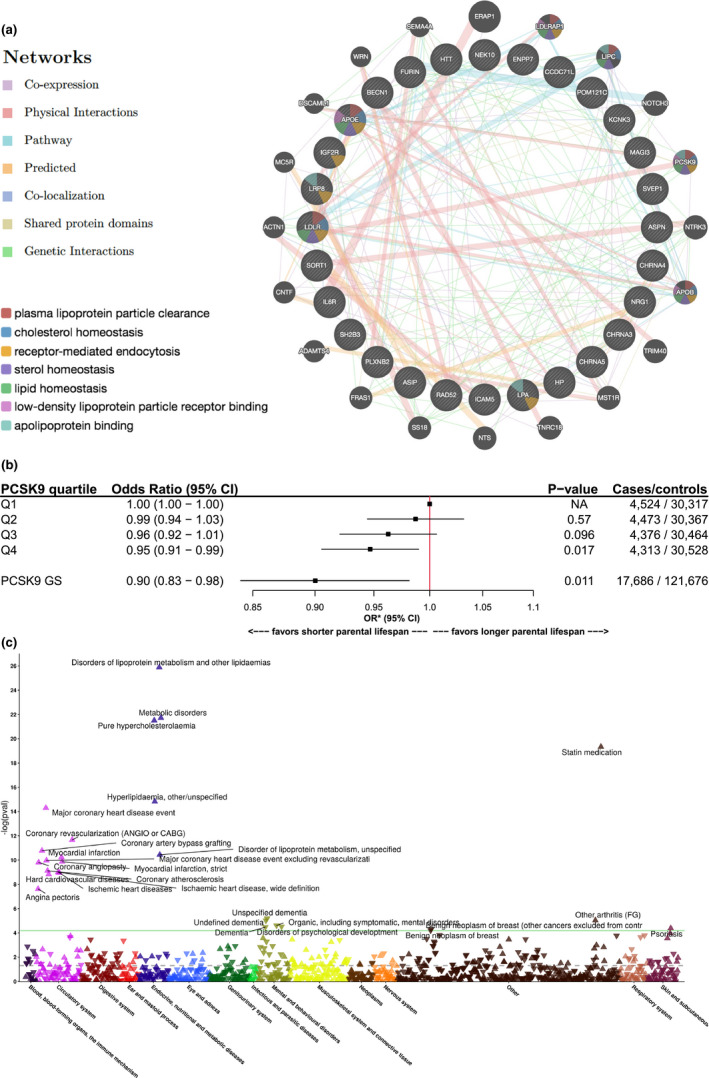
Investigation of proprotein convertase subtilisin/kexin type 9 (PCSK9) inhibition as a potential therapy for chronic diseases. (a) Interaction network of parental lifespan eGenes and eProteins with other genes and top pathways of parental lifespan eGenes and eProteins (false discovery rate <5e^−5^) from the GeneMANIA prediction server. (b) Odds ratio (OR) and 95% confidence interval (CI) for high parental lifespan in participants of the UK Biobank separated into quartiles of the *PCSK9* genetic score (GS) of 11 independent variants associated with low‐density lipoprotein cholesterol levels. Analyses were adjusted for age, sex and 10 first ancestry‐based principal components. The adjusted odds ratio and 95% CI for high parental lifespan, associated with a 1 mmol/L increase in low‐density lipoprotein cholesterol associated with these variants is also shown. (c) Phenome‐wide inverse variance weighted Mendelian randomization study depicting the association between variants at the *PCSK9* locus weighted for their impact on low‐density lipoprotein cholesterol and 1169 binary disease‐related traits in FinnGen. Associations are reported after correction for multiple testing, which accounted for phenotypic correlation between traits. Arrows pointing up represent higher disease presence and arrows pointing down represent lower disease presence. The dotted line represents the nominal *p*‐value of 0.05 and the green line represents the *p*‐value after correction for multiple testing

As many of the eGenes, eProteins and eMetabolites were associated with age‐related chronic disease, we aimed at identifying in a more comprehensive manner which disease traits were influenced by these exposures. We performed a systematic analysis of potential diseases associated with eGenes and genes encoding eProteins in the Open Targets Platform (Carvalho‐Silva et al., 2019). eMetabolites were not considered in this analysis as their variance is determined by more than one gene each contributing to a different extent to eMetabolite levels. Table S9 presents the diseases and therapeutic areas relevant to the identified eGenes and eProteins with *p*‐values <5e^−8^ for association score with therapeutic areas concordant with the effect of eGenes and eProteins (Table 1 and Figure 2) on chronic diseases. We further searched the Open Targets Platform to identify drug candidates targeting eGenes and eProteins. Table S10 presents 23 drug candidates that were identified by Open Targets Platform. These drugs target four parental lifespan eGenes (*CHRNA4*, *KCNK3*, *CHRNA3*/*5*, and *HTT*) and one parental lifespan eProtein (*IL6R*). A second strategy was also used to intersect eGenes and eProteins with the DrugBank and the Drug Gene Interaction database (Wishart et al., 2018). This analysis identified new targets potentially interacting with parental lifespan eGenes and eProteins (Tables S11 and S12). Finally, we used the network processing data tool GeneMANIA to identify new genes that may show co‐expression or physical interaction with our targets (Franz et al., 2018). The inner circle of Figure 4a includes the eGenes and genes encoding for eProteins that we have entered in the engine while the outer circle presents new targets that show co‐expression or physical interaction with our targets. Consistent with Timmers et al. (2019), this analysis enabled the identification of the top biological pathways (all with FDR *p*‐value < 1e^−4^), which are all related to lipoprotein metabolism. Proprotein Subtilisin/Kexin Type 9 (PCSK9) was identified in the biological pathway analysis as interacting with several of the parental lifespan associated eGenes such as the LDLR. Through this strategy, we also identified PCSK9 inhibitors as potential lifespan extending drug candidates.

### 2.6 Genetic investigation into PCSK9 as a therapeutic target for chronic diseases

PCSK9 emerged as a potential drug target for several reasons. PCSK9 is involved in the top 6 pathways suggested by GeneMANIA. This tool also suggested co‐expression with *LDLR* (another parental lifespan locus) and experimental evidence supports this finding since both PCSK9 and the LDLR are under sterol regulatory element‐binding protein 2 control, a regulatory point in cholesterol metabolism (Rashid et al., 2005). PCSK9 interacts with apolipoprotein B, and an important proportion of circulating PCSK9 is carried by apolipoprotein‐B‐containing lipoproteins (Kosenko et al., 2013) including lipoprotein(a), another parental lifespan locus, *LPA* (Tavori et al., 2016). PCKS9 also has shared protein domains with another parental lifespan eGene, *FURIN* (also supported by experimental evidence since furin has the ability to cleave PCSK9 (Benjannet et al., 2006)). DrugBank and the Open Targets Platform suggested PCSK9 neutralizing antibodies (alirocumab and evolocumab) as potential drug repurposing opportunities. Finally, PCSK9 inhibitors have important LDL cholesterol reduction properties (up to 60%). As our investigation reported a strong and causal effect of exposure to low LDL cholesterol levels and parental lifespan (Figure 2b), we investigated the association between genetic variation at the *PCSK9* locus and parental lifespan using individual participant level data in the UK Biobank. We identified 11 independent SNPs within the *PCSK9* region strongly and independently associated with LDL cholesterol in the Global Lipids Genetic Consortium (*r* ^2^ < 0.1 and *p*‐value < 5e^−8^). We evaluated the impact of carrier status of *PCSK9* SNPs linked with high LDL and the impact of a weighted genetic score (GS) scaled to model a 1 mmol/L increase in LDL‐C levels and found a significant association with having lower odds of high parental lifespan (Figure 4b). We next performed a phenome‐wide MR analysis across the disease‐related phenome to gain more insight on the potential benefits/risk ratio of PCSK9 inhibitors. In this phenome‐wide MR, the association between 11 SNPs at the *PCSK9* locus (weighted for their impact on plasma LDL cholesterol levels) and 1169 binary disease‐related traits in 135,638 participants of the FinnGen cohorts was investigated using IVW‐MR. As expected, PCSK9 “genetic inhibition” may target aging‐associated diseases through its association with lifelong exposure to low LDL cholesterol levels and associated reduction in the risk of cardiovascular diseases (Figure 4c and Table S13).

### 2.7 Mendelian randomization studies across the human phenome

We used PhenomeXcan (Pividori et al., 2020), a resource enabling the investigation of eGenes across the human phenome, to identify human traits and diseases than may be causally influenced by parental lifespan eGenes. This analysis revealed 669 eGene‐trait associations of interest after correction for multiple testing (Table S14). We observed noteworthy associations such as links between *LRP8* and psychological traits, *SORT1* and cholesterol metabolism, *KCNK3* and hypertension and obesity, *NEK10* and cardiovascular traits, *HTT* and platelet count, alcohol intake, socio‐economic status and obesity, *POM121C* and obesity, *CCDC71L* and platelet count and hypertension, *NRG1* and height, *RAD52* and basal metabolic rate and obesity, *CHRNA2*/*3*/*4*/*5* and smoking behavior and lung phenotypes, *FURIN*/*FES* and cardiovascular traits, *HP* and lipid and hemoglobin concentrations as well as *BECN1* and abdominal obesity. The eGenes, eProteins and eMetabolites that showed robust association with our longevity trait could represent potential therapeutic targets for other human diseases. In order to gain knowledge on the function of these genes and proteins, and to predict potential benefits and/or adverse effects of therapeutic agents developed to mimic the effect of eGenes, eProteins eMetabolites on human health, we performed a phenome‐wide MR across a broad range of human diseases. We used IVW‐MR with genetic instruments for eGenes, eProteins and eMetabolites to investigate 1169 disease‐specific binary traits in the FinnGen cohorts. Parental lifespan genome‐wide significant variants not having TWAS associations (*MAGI3*, *CDKN2B*‐*AS1*, *LPA*, *LDLR* and *APOE*), were also included in this analysis, the exception being *ATXN2*/*BRAP*, which we considered an eGene (*SH2B3*) as this gene passed the TWAS significance threshold with posterior probability of genetic colocalization of 0.63. Details on all diseases that passed correction for multiple testing using MR‐pheWAS analyses in FinnGen are presented in Tables S15, S16, S17 and S18 for parental lifespan eGenes, eProteins, eMetabolites and other parental lifespan SNPs, respectively. This analysis yielded 270 eGene‐disease trait associations, 239 eProtein‐disease trait associations, 57 eMetabolite‐disease‐trait association and 223 parental lifespan SNP‐disease trait associations. Thus, targeting some of the parental lifespan eGenes, eProteins or eMetabolites may prevent age‐associated diseases such as cardiovascular diseases and type 2 diabetes and the risk factors for these diseases such as smoking, blood cholesterol levels, inflammation, obesity and hypertension.

## 3 DISCUSSION

Our translational approach combined TWAS with genetic colocalization, proteome‐wide, metabolome‐wide and phenome‐wide MR seeking to identify novel candidate biological determinants of parental lifespan and their relevance in age‐associated human diseases. The newly identified genomic loci colocalizing with parental lifespan include *LRP8*, the gene encoding the low‐density lipoprotein receptor‐related protein 8 (also known as ApoER2), *NEK10*, the gene encoding NIMA related kinase 10, *CCDC71L* the gene encoding Coiled‐coil domain‐containing protein 71L), *NRG1*, the gene encoding neuregulin‐1, *RAD52*, the gene encoding DNA repair protein RAD52 homolog and *BECN1*, the gene encoding beclin‐1, the latter being reported in our previous MR study of parental lifespan using blood gene expression as instrumental variables) (Chignon et al., 2020). Our approach strengthens the case for previously identified regions by GWAS by the identification of a tissue‐specific colocalized TWAS signal for the *KCNK3*, *ADD1*/*GRK4*/*HTT*, *HP*/*HPR*/*TXNL4B*, *LAMA5*/*AL121832*.*2*/*CABLES2*/*CHRNA4* loci. We provide evidence using genetic colocalization that genetic regions previously identified by TWAS (*CELSR2*/*PSRC1*/*SORT1*, *POM121C*, *CHRNA3/5* and *FES*/*FURIN*) are likely causally associated with parental lifespan. Results of our analysis also suggest tissue‐specific regulation of parental lifespan eGenes consistent with a biological effect. Some examples include brain eGenes such as *LRP8* and smoking‐associated eGenes (*CHRNA2*/*3*/*4*/*5*), liver eGenes such as *HP* and *SORT1*, and a skeletal muscle eGene (*HTT*). Finally, we provide evidence that nine circulating proteins (haptoglobin [*HP*], asporin [*ASPN]*, agouti‐signaling protein [*ASIP]*, the soluble receptor of insulin‐like growth factor 2 [*IGF2R]*, plexin‐B2 [*PLXNB2]*, interleukin‐6 receptor subunit alpha [*IL6R*], soluble intercellular adhesion molecule‐5 [*ICAM5*], ectonucleotidase phosphodiesterase 7 [*ENPP7*] and Sushi, von Willebrand factor type A, EGF and pentraxin domain‐containing protein 1 [*SVEP1*] may be causally associated with parental lifespan. Many of these blood factors could represent therapeutic targets to reduce the risk of age‐associated chronic diseases and longevity. Our search for parental lifespan eMetabolites revealed that circulating metabolites such as amino acids or energy metabolism associated molecules may not be causally associated with parental lifespan. However, plasma levels of apolipoprotein‐B‐containing lipoproteins (VLDL and LDL) were strongly associated with shorter parental lifespan. This MR analysis provides genetic confirmation of the results of several investigations into the long‐term health benefits of lipid‐lowering therapies (Collins et al., 2016; Pedersen, 2016).

In support of the causal association of apolipoprotein‐B‐containing lipoprotein and parental lifespan, we found through a gene interaction network that the four genes responsible for the overwhelming majority of the cases for the most prevalent human genetic disease (Defesche et al., 2017), familial hypercholesterolemia, could be linked with genes regulating lifespan (*LDLR*, *PCSK9*, *APOB* and *LDLRAP1*). These results support the notion that lifelong exposure to low concentration of all apolipoprotein‐B‐containing lipoprotein particles might be an important determinant of atherosclerotic coronary artery diseases and human longevity. In this work, we also provide evidence that genetic variation in *PCSK9* mimicking the effect of PCSK9 inhibitors is linked to lifelong exposure to low LDL cholesterol levels and higher parental lifespan. These results suggest that PCSK9 inhibitors, by reducing LDL cholesterol, could be key to decreasing aging‐associated diseases and extending lifespan but need to be tested in adequately powered long‐term randomized clinical trials.

A non‐colocalized signal was also reported for a liver expressed gene: *LPA*, the gene that encodes apolipoprotein(a), which is one of the components of lipoprotein(a) (Lp[a]), another apolipoprotein‐B‐containing highly atherogenic lipoprotein particle (Tsimikas et al., 2018). We have previously reported in a hypothesis‐driven MR study a strong relationship between plasma Lp(a) levels and parental lifespan, healthspan (defined as disease‐free survival) and long‐term mortality risk (Arsenault et al., 2020). Long‐term health outcome trials of lipoprotein(a)‐lowering therapies with antisense oligonucleotides are currently underway (NCT04023552).

In their landmark study investigating the genetics of parental lifespan in more than one million individuals, Timmers et al. (2019), developed and used a new approach called Bayesian prior‐informed GWAS. This method identifies new genetic variants associated with parental lifespan based on mortality risk factors. This approach led to the discovery of 7 loci associated with parental lifespan. Our trans‐omic MR study identified 3 of these loci (without using prior information on mortality risk factors): *IGF2R* using proteome‐wide MR as well as *POM121C* and the *CELSR2*/*PSRC1*/*SORT1* region using TWAS. Although the same genetic variant was responsible for the genetic colocalization of the three co‐regulated genes at the 1p13 locus (*CELSR2*/*PSRC1*/*SORT1* region), *SORT1* might be responsible for the parental lifespan signal at the 1p13 locus. Indeed, a large body of evidence supports a functional impact of sortilin (encoded by the *SORT1* gene) in lipoprotein metabolism (Kjolby et al., 2010; Musunuru et al., 2010). Our findings add novel information on the regulation of genes that may be linked with parental lifespan. Although Timmers et al. provided evidence that some cis‐acting variants associated with parental lifespan did influence gene expression, a TWAS followed by genetic colocalization investigating potentially new eGenes associated with parental lifespan was not performed as we report here. We also linked the genetically regulated genetic expression of these genes with several cardiometabolic traits as well as human diseases. Altogether, these results support the usefulness of combining various genetic investigation techniques relying on mortality risk factors and MR to identify new genetic and biological determinants of complex traits such as human longevity.

Results of this study further confirm the role of smoking behaviors as a strong determinant of lifespan and chronic diseases. We found that many smoking‐associated loci such as cholinergic receptor nicotinic 2, 3/5 and 4 subunits may actually be brain eGenes. Timmers et al. (2019) did identify variation at *CHRNA 3*/*5* on chromosome 15 to be associated with parental lifespan at the genome‐wide significance level. Here we report that *CHRNA4* on chromosome 20 and *CHRNA2* on chromosome 8 as new potential parental lifespan loci (although the latter did not show evidence of genetic colocalization). Unsurprisingly, our search for potential drug targets for aging‐associated diseases identified many smoking cessation therapies and the results of our phenome‐wide MR studies confirmed the association of these eGenes with atherosclerotic cardiovascular diseases and neoplasms. These results confirm the importance of urgently adopting strict tobacco control policies throughout the world to improve global health, decrease chronic disease burden and human longevity.

In this analysis, the only protein that showed significant association in genome‐wide, transcriptome‐wide and proteome‐wide MR studies was haptoglobin (Hp). We show here that *HP* is a liver expressed gene and that liver expression of *HP*, as well as plasma Hp is linked with parental lifespan. A study showed that Hp levels are higher in the plasma of Japanese semisuper centenarians (Miura et al., 2011). Hp is an acute‐phase protein and is one of the most abundant proteins in human plasma. It binds free hemoglobin and facilitates its removal from the bloodstream. Our analysis also identified an association between genetically regulated *HP* genetic expression and hemoglobin levels. Hp also decreases oxidation of apolipoprotein E (encoded by the longevity gene *APOE*). A previous study showed that exon deletion in *HP* was associated with lower LDL cholesterol levels (Boettger et al., 2016). The authors proposed a mechanism whereby Hp reduced the oxidation of apolipoprotein E and facilitated its removal from the blood stream, thereby reducing LDL cholesterol levels. Upon further investigation of the *HP* locus on chromosome 15, we found strong evidence of genetic colocalization between liver *HP* expression, LDL‐C levels and parental lifespan, but not plasma Hp levels. We believe that this finding might be due to the fact that the assessment of plasma Hp levels may not capture the full extent of the various circulating Hp isoforms (1–1, 1–2 and 2–2). Interestingly, our gene network analysis revealed a potential interaction of *HP* and *APOE*. Interaction of Hp, APOE and beta‐amyloid was also reported in human brain tissues, where this complex might influence central cholesterol metabolism (Spagnuolo et al., 2014). This finding is also supported by our phenome‐wide MR study in FinnGen reporting a positive association between liver expression of *HP* (but not blood levels of Hp) and Alzheimer's Disease. Functional genomics and experimental investigations will be needed to shed light on the mechanisms linking *HP* and longevity and to determine if *HP* might represent a potential therapeutic target to improve lifespan.

The majority of previous investigations into the biological determinants of aging have contributed to the identification of several biological mechanisms regulating longevity in model organisms. These mechanisms include, among others, the modulation of the insulin‐like signalling pathway, target of rapamycin, sirtuins and NAD+, circadian clocks, mitochondria and oxidative stress, senescence, chronic inflammation, autophagy, proteostasis as well as genomic instability and telomere attrition (Campisi et al., 2019). Although the biological determinants of aging in humans are still incompletely understood, aging mechanisms identified in model organisms appear to contrast with the biological determinants of aging in humans. One exception appears to be chronic inflammation, which might influence longevity in both model organisms and humans. Both our recent report of genetic variation in the interleukin (IL)‐6 signalling pathway being associated with parental lifespan (Rosa et al., 2019) and the results of the CANTOS trial, which reported important mortality benefits in patients with elevated high‐sensitivity C‐reactive protein levels following administration of the IL‐1β monoclonal antibody canakinumab (Ridker et al., 2018) support this hypothesis. Our study builds on this previous work showing that genes involved in both innate and immune response may be associated with longevity (Chignon et al., 2020). Here, our proteome‐wide MR study identified variation at the *IL6R* and *ICAM5* loci to be associated with parental lifespan (although evidence of horizontal pleiotropy in the IL6R‐parental lifespan association was detected). Although some cardiovascular benefits could be observed by targeting the IL‐6 or ICAM5 pathways, our phenome‐wide MR results suggest that this might come at the price of increased risk of atopic disorders.

Other noteworthy findings of our study include a possible interaction between a newly identified locus, *RAD52* and *WRN*, the Werner syndrome ATP‐dependent helicase. Coordination of these two proteins activities involves telomere metabolism through their action on replication fork rescue after DNA damage in cells of patients with Werner syndrome (a premature aging disorder). Another finding of interest is the identification of Sushi, von Willebrand factor type A, EGF and pentraxin domain‐containing protein 1 (SVEP1) as a circulating protein causally associated with parental lifespan. Interestingly, a previous analysis also reported a potential association in this gene with longevity in the Framingham Heart Study (Yashin et al., 2010). Finally, the association of *CCDC71L* expression in arteries with shorter parental lifespan and higher risk of coronary artery disease in FinnGen also warrants further exploration.

The results of this study need to be interpreted in the context of certain limitations of our approach, beginning with the definition of the study outcome. By definition, parents have to reach their reproductive age to be included (i.e., have children that were enrolled in the study cohorts), which may introduce selection bias. Although efforts were made to exclude relatives as well as parents that died before the age of 40, the cause of parental death was not available. Parents were also exposed to a different environment than their children (exposure to world wars, famine, higher rate of smoking, heterogeneous living conditions, sanitation and access to medical care, etc.). This observation, combined with the fact that parental lifespan is a self‐reported trait, may have introduced misclassification. The effect sizes of the variants that were used to assess our study exposures (eGenes, eProteins and eMetabolites) were obtained in modern cohorts from individuals not necessarily exposed to such conditions and it is unsure if these could have influenced the effect of these variants on our exposures of interest. We were not able to replicate the effect of genetic variants on study exposures in a second cohort as, to our knowledge, only GTEx provides enough variation on common variants with effect sizes on gene expression levels across such as wide range of tissues. For eProteins, although other datasets are available, most report only top pQTLs and the summary statistics are not available. Therefore, whether the therapeutic targets identified in this study will help extend lifespan of future generations need to be validated and replicated in additional genetic association studies from modern cohorts and more importantly, in prospective randomized clinical trials. Finally, although several methods were used to correct for false positives and spurious gene prioritization (genetic colocalization) with regards to the eGene analysis and to control for horizontal pleiotropy and outliers for eProteins and eMetabolites (use of multiple Mendelian randomization method), these methods are not definitive, especially since we could not validate these results in an independent cohort.

In conclusion, our study identified new genetically regulated genes across 43 tissues, as well as genetically regulated circulating proteins and metabolites that could potentially regulate human lifespan. Many of these genetic determinants of parental lifespan represent potential therapeutic targets for aging‐associated diseases. Our study also underscores the importance of global population health measures such as adopting stricter tobacco control measures as well as the globalization of interventions targeting all apolipoprotein‐B‐containing particles to prevent the onset of diseases of the cardiovascular system and possibly promote longevity.

## 4 METHODS

### 4.1 Multi‐tissue transcriptome‐wide association study of parental lifespan

The main study outcome was parental survival, which was obtained from summary statistics of the study of Timmers et al. (2019) who performed a GWAS of survival in a sample of 1,012,240 parents from the UK Biobank (691,621 parental lifespans) and cohorts of the LifeGen consortium (320,619 parental lifespans from 26 additional population cohorts). This study identified parental lifespan was defined by the age of parent death or dead/alive status. Parents with an age of death <40 were excluded. The association test was conducted under the Cox's proportional hazards model, which implies a positive effect size for a longer life. Transcriptome‐wide association studies (TWAS) integrate GWAS and eQTLs to identify genes with a genetically regulated level of expression (eGenes) associated with human quantitative traits and/or diseases. In an attempt to implicate eGenes in the etiology of parental lifespan across multiple tissues, Timmers et al. (2019) used Summary‐level Mendelian Randomization and Heterogeneity in Independent Instruments (HEIDI) tests from eQTL mapping in 48 tissues of the GTEx consortium. Although this approach helped identify the causal gene implicated in parental lifespan from the main GWAS signal, few if any new longevity genes were identified using TWAS. We used data from the Genotype‐Tissue Expression Project (GTEx) resource (version 8) to perform TWAS on parental lifespan. GTEx is a large‐scale multi‐omic dataset where DNA and RNA were collected postmortem from 53 tissue samples from 635 donors. Tissues with less than 70 samples were removed to provide sufficient statistical power for eQTL discovery, resulting in a set of 48 tissues. Only non‐sex‐specific tissues (*N* = 43) were analyzed. Alignment to the human reference genome hg28/GRCh38 was performed using STAR v2.6.1d, based on the GENCODE v30 annotation. RNA‐seq expression outliers were excluded using a multidimensional extension of the statistic described by Wright et al. (2014) Samples with less than 10 million mapped reads were removed. For samples with replicates, replicate with the greatest number of reads were selected. Expression values were normalized between samples using TMM as implemented in edgeR (Robinson & Oshlack, 2010). For each gene, expression values were normalized across samples using an inverse normal transformation. Gene‐level analyses were performed with S‐PrediXcan (Barbeira et al., 2018), which estimates cis‐eQTL effect sizes with reference datasets. Resulting eQTL effects sizes were used to impute eGenes that were tested using summary statistics of the parental lifespan GWAS described above. S‐PrediXcan enables TWAS without the need of individual‐level data based on the MetaXcan framework. S‐PrediXcan uses a parametric model (elastic net) that assumes a combination of LASSO and Ridge penalties on the eQTL effect sizes. eQTL prediction models were performed using elastic net, a regularized regression method, as implemented in the PredictDB pipeline (Barbeira et al., 2018; Gamazon et al., 2015). We used SNPs with a minor allele frequency greater than 1% from European ancestry participants. Expression of protein coding, antisense, long intergenic non‐coding and micro RNA was considered. The first three ancestry‐based principal components, a set of covariates identified using the probabilistic estimation of expression residuals method (Stegle et al., 2012) with genotyping platform and sex were used as covariates.

### 4.2 Assessment of genetic colocalization

Genetic colocalization was used to filter our LD‐contaminated associations. A stringent Bayesian model is used to estimate the posterior probability of each eGene containing a single eQTL affecting both the eGene and the outcome(s) of interest. We used COLOC R package to estimate the probability of five hypotheses: H0 corresponds to no eQTL and no GWAS association, H1 and H2 correspond to eQTL association but no GWAS association or vice‐versa, H3 corresponds to eQTL and GWAS associations but independent signals (weaker eQTL signal or GWAS hit) and H4 corresponds to shared eQTL and GWAS signal (the lead eQTL is also among the top GWAS hits) (Giambartolomei et al., 2014). We kept eGenes with evidence of genetic colocalization (PP.H4 > 0.75). To assess the role of the prior, we varied the critical parameter *p12*, which codes for the prior probability that a variant is associated with the exposure and the outcome (1e−4, 1e−5 and 1e−6). For cis‐eQTL analyses, a *p*_12_ = 1e−4 threshold is usually suggested. Table 1 presents the PPH4 results with the prior set at the more conservative threshold of *p*_12_ = 1e−5. *Locuscompare* function from the *LocuscompareR* R package (Liu et al., 2019) was used to depict the colocalization events. This software enables visualization of the strengths of eQTLs and outcomes associations by plotting *p*‐values for each within a given genomic location, thereby contributing to distinguish candidates from false‐positive genes. To account for multiple testing, a Bonferroni correction for all gene‐tissue pairs: <0.05/number of gene‐tissue pairs tested was applied.

### 4.3 Associations of eGenes with parental lifespan using Mendelian randomization

The association of eGenes linked with parental lifespan in TWAS analysis and parental lifespan was investigated using GTEx and eQTLGen. We used IVW‐MR with the *mr* function from *TwoSampleMR* package. The IVW‐MR is comparable to performing a meta‐analysis of each Wald ratio. Additional MR analyses were performed to evaluate heterogeneity (intercept *p*‐value, from MR Egger (Bowden et al., 2017)) and the presence of outliers. We used MR‐PRESSO (Verbanck et al., 2018), an outlier robust method, to detect the presence of outliers (variants potentially causing pleiotropy and influencing causal estimates) and causal estimates were obtained before and after excluding outliers. We also used the contamination mixture method as this method was recently identified as a robust modelling method to identify causal relationships in the presence of potentially invalid genetic instruments (Burgess et al., 2020).

### 4.4 Tissue specificity of gene expression

The tissue‐specific gene expression metric (τ) was obtained from all genes identified by TWAS. We used the formula from Yanai et al. (2005) to compare the level of gene expression across selected tissues based on RNA sequencing data from European ancestry donors from GTEx. All the genes with expression <1 RPKM were set as not expressed. The RNA‐seq data were first log‐transformed. After the normalization, a mean value from all replicates for each tissue separately was calculated. A τ value closer to 1 indicates tissue specificity while a τ value closer to 0 indicates ubiquitous gene expression. We considered that eGenes had tissue‐specific expression when their τ statistic was ≥0.80.

### 4.5 Associations of eProteins and eMetabolites with parental lifespan

We used GWAS summary statistics from the INTERVAL cohort (Sun et al., 2018) to identify eProteins that could potentially be causally associated with parental lifespan. Relative concentrations of 3,622 plasma proteins or protein complexes were assayed using an aptamer‐based multiplex protein assay (SOMAscan) in 3,301 participants from the INTERVAL study. A minimum of 4 instrumental variables (IVs) were constructed using cis‐pQTLs in a 1Mb window, clumped using *plink 1*.*9* with a *r* ^2^ < 0.1 (from 1000 genomes phase 3 European ancestry LD reference panel), a *p*‐value <5e−8, and a physical distance threshold of 250 Kb. The MHC (6:28,477,797–33,448,354) and APOE regions were removed as well as ABO gene and immunoglobulins. Association of 279 circulating eProteins and parental lifespan was assessed with IVW‐MR using *mr* function from *TwoSampleMR* package and a Bonferroni correction was applied (*p*‐value = 1.79e^−4^ (0.05/279 eProteins). Additional MR analyses were performed to evaluate heterogeneity (MR Egger (Bowden et al., 2017)) and the presence of outliers (MR‐PRESSO (Verbanck et al., 2018)). We also used the contamination mixture method (Burgess et al., 2020). The same MR framework was used to identify eMetabolites potentially associated with parental lifespan. We used GWAS summary on 123 metabolites from the study of Kettunen et al. (2016) In this study, 123 blood lipids and metabolites were measured in 24,925 individuals from 10 European cohorts using high‐throughput nuclear magnetic resonance spectroscopy. Metabolites measured by NMR represent a broad molecular signature of systemic metabolism and includes metabolites from multiple metabolic pathways (lipoprotein lipids and subclasses, fatty acids as well as amino acids, glycolysis precursors, etc.). IVs for eMetabolites were constructed using independent genetic variants clumped using *plink 1*.*9* with a *r* ^2^ < 0.1, a *p*‐value <5e−8 and a physical distance threshold of 250 Kb. Association of 115 circulating eMetabolites and parental lifespan was assessed with IVW‐MR using *mr* function from *TwoSampleMR* package and a Bonferroni correction was applied (*p*‐value = 4.35e^−4^ (0.05/115 eMetabolite). MR‐PRESSO, MR‐Egger and contamination mixture methods were also used as described above.

### 4.6 Investigation of genetic colocalization across multiple traits at the HP locus

We investigated genetic colocalization across multiple traits at chromosome 15 within 1 Mb of the *HP* gene between liver expression of the *HP* gene (from GTEx (Lonsdale et al., 2013)), plasma Hp levels (from INTERVAL (Sun et al., 2018)), plasma LDL‐C levels (from GLGC (Willer et al., 2013)) and parental lifespan (from UK Biobank and LifeGen (Timmers et al., 2019)) using the *hyprcoloc* R package. HyPrColoc is a deterministic Bayesian algorithm using GWAS summary statistics that can detect colocalization across vast numbers of traits simultaneously (Foley et al., , 2021). Posterior probabilities of colocalization heatmap was performed with the *sensitivity*.*plot* function from the *hyprcoloc* R package with default settings (regional and alignment thresholds: 0.6, 0.7, 0.8 and 0.9, prior probabilities of colocalization: 0.98, 0.99 and 0.995). Regional association plot was obtained from the *stack_assoc_plot* function from the *gassocplot* R package and the 1000G phase 3 LD reference panel (European ancestry).

### 4.7 Resources used to identify drug candidates for aging‐associated diseases

We used the Open Targets Platform to determine the potential therapeutic areas of the eGenes and eProteins associated with parental lifespan and to identify approved therapies that may target these eGenes or eProteins. Briefly, the Open Targets Platform developed a user‐friendly web interface (available at: https://www.targetvalidation.org/) that enables the search of therapeutic areas, drug targets, pathways, gene ontology, and tractability information simultaneous for gene lists prepared by the user. This information is made available by the platform's integration of evidence from genetics, genomics, transcriptomics, drugs, animal models and scientific literature to score and rank target‐diseases associations for drug target identification. We also used the DrugBank encyclopedia to identify potential drug repositioning opportunities and drug targets that may interact with eGenes and eProteins identified in the course of this investigation. DrugBank is a publicly available resource with drug and drug target information on over 13,000 drug entries available at: https://www.drugbank.ca/. The Drug Gene Interaction database is an open source resource than enables the query of genes of interest with respect to known drug gene interactions and potential druggability, it is available at: http://www.dgidb.org/ (Cotto et al., 2018). We also queried eGenes and eProteins in the GeneMANIA prediction server to gain new information on pathways and genetic interactions mostly connected with the eGenes and eProteins (Warde‐Farley et al., 2010). Briefly, GeneMANIA integrates data from thousands of genomics and proteomics datasets such as the Gene Expression Omnibus (GEO), BioGRID, IRefindex, I2D as well as organisms‐specific functional genomics datasets to enable users to query list of genes in a user‐friendly web portal. GeneMANIA algorithms use patterns of gene co‐annotations in the Gene Ontology (GO) biological functions hierarchy, which are weighted to find closely connected genes and generates networks of gene co‐expression, proteins with physical interactions, shared domains, etc.

### 4.8 Variation in PCSK9 and parental lifespan using individual participant data in the UK Biobank

We selected independent SNPs (*r* ^2^ < 0.10) at the *PCSK9* locus (within 100 Kb of the gene) associated with LDL‐C levels at a genome‐wide level of significance in the Global Lipids Genetics Consortium (GLGC) (Willer et al., 2013). This approach yielded 11 SNPs independently associated with LDL‐C levels. We constructed a weighted genetic score (GS) of SNPs at the *PCSK9* locus scaled to a 1 mmol/L reduction in LDL‐C levels. The associations between the GS and parental lifespan in the UK Biobank was tested using logistic regression adjusted for age, sex and the first 10 ancestry‐based principal components using R (version 3.5.1). We used the definition of Pilling et al. (2017) to assess high parental lifespan in participants of the UK Biobank. Only participants between 55 and 69 years were included. Participants who were adopted, had missing information on age of parents’ death or who had parents who died at a young age (<46 for the father and <57 for the mother) were excluded from these analyses. Parents were separated into three categories: long‐lived (father still alive and older than 90 or father's age of death ≥90 and mother still alive and older than 93 or mother's age of death ≥93), medium‐lived (age of death ≥66 and <89 for the father and ≥73 and <92 for the mother) and short‐lived (age of death ≥46 and <65 for the father and ≥57 and <72 for the mother). We defined high parental lifespan as at least one long‐lived parent (i.e., long/long or long/medium). The control group included participants with parents considered as short‐ or medium‐lived (i.e., short/short, short/medium or medium/medium). Participants discordant for mothers' and fathers' age of death (one long‐lived parent and one short‐lived parent) were also excluded from the present analyses. These analyses were conducted under UK Biobank data application number 25205.

### 4.9 Effect of parental lifespan eGenes on other traits and human disease

The association between parental lifespan eGenes (presented in Table 1) and human traits and disease was investigated using PhenomeXcan (Pividori et al., 2020). PhenomeXcan is a queryable platform that combines GWAS summary statistics on 4091 human traits and transcriptome‐wide expression data on 22,515 genes in 49 tissues from GTEx v8. We interrogated results from the Summary MultiXcan platform, an analytical method that uses summary statistics and aggregate results across tissues. We present parental lifespan eGene‐trait associations with a *p*‐value <1.2e^−5^ (0.05/4091 traits). The fastENLOC‐assessed regional colocalization probability (RCP) is also presented.

### 4.10 Association of eGenes, eProteins, eMetabolites and PCSK9 with disease traits in FinnGen

For MR analysis, cis‐eQTL were identified using QTLtools (Delaneau et al., 2017) with the first three principal components, a set of covariates identified using the probabilistic estimation of expression residuals method (Stegle et al., 2012) with genotyping platform and sex as covariates. Variants were included if they had a minor allele frequency ≥1%. Missing genotypes were imputed as the mean of the other participants' genotypes. The cis‐window size was set to 1Mb. We used the *get_r_from_pn* and *get_r_from_lor* functions from the *TwoSampleMR* package to calculate variance of each eQTL for quantitative and binary data respectively. SNPs that explain more of the variance in the outcome compared to the exposure was not included as instrumental variables (IV). Removing eQTLs that show evidence of reverse causality is of particular importance in the setting of human longevity studies as aging might influence genes in a tissue‐specific manner (Yang et al., 2015). IVs were clumped using plink 1.9 with a *r* ^2^ < 0.1, a *p*‐value <0.01 and a physical distance threshold of 250 Kb. If an eGene was significant in multiple tissues, the tissue that provided the strongest eGenes‐parental lifespan estimate from S‐PrediXcan was prioritized. Instruments for eProteins and eMetabolites were the same as those used to determine their association with parental lifespan. In FinnGen, a method called SAIGE (Scalable and Accurate Implementation of Generalized Mixed Models), which is based on generalized mixed models was developed to control for case‐control imbalance, sample relatedness and population structure. GWAS was performed using over 16 million genetic markers genotyped with the Illumina or Affymetrix arrays or imputed using the population specific SISu v3 reference panel. Variables included in the models were sex, age, the 10‐main ancestry‐based principal components and genotyping batch. This analysis was performed using publicly available GWAS summary statistics from the FinnGen data freeze 3 (June 16, 2020). Outcomes with <400 cases were excluded leaving 1169 traits for PheWAS. Since several of the phenotypes that were investigated were genetically correlated, accounting for all phenotypes as they were independent may be too conservative. We therefore used the PhenoSpD tool (Zheng et al., 2018), to estimate the number of independent tests that are performed. PhenoSpD applies GWAS summary statistics to LD score regression to estimate the phenotypic correlation matrix of the traits and estimates the number of independent variables among the traits. We considered associations that had a *p*‐value <6.5e^−5^ (0.05/773 traits) (instead of 1169) to be statistically significant. We used IVW‐MR to determine the association between genetic instruments for eGenes, eProteins, eMetabolites, single SNPs without TWAS (*APOE*, *LPA*, *LDLR*, *MAGI3* and 9p21 region) or LDL‐C reductions associated with variants at the *PCSK9* locus with these disease‐specific binary traits. We used genetic instruments mentioned above while keeping the SNPs in common between the compared datasets.

## CONFLICT OF INTEREST

B.J.A. is a consultant for Silence Therapeutics and Novartis and has received research funding from Ionis Pharmaceuticals, Pfizer and Silence Therapeutics.

## AUTHOR CONTRIBUTIONS

N.P., W.P., J.B., C.C. and Z.L. analyzed the data, N.P., C.C. and B.J.A. made the figures, N.P. and B.J.A. drafted the manuscript. All the others interpreted the data and critically reviewed the manuscript prior to submission.

## PATIENT CONSENT STATEMENT

All contributing datasets obtained patient consent.

## Supporting information

Table S1

Table S2

Table S3

Table S4

Table S5

Table S6

Table S7

Table S8

Table S9

Table S10

Table S11

Table S12

Table S13

Table S14

Table S15

Table S16

Table S17

Table S18

Supplementary Material

## Data Availability

The summary statistics of the GWAS meta‐analysis of parental lifespan are available for download at https://datashare.is.ed.ac.uk/handle/10283/3209. GTEx data is available to download at https://gtexportal.org/home/datasets. The data used for the analyses described in this manuscript were obtained from dbGaP, accession number phs000424.vN.pN. Data of the eQTLGen consortium is available at https://www.eqtlgen.org/. The GWAS summary statistics for >1,800 binary phenotypes in the FinnGen cohorts by SAIGE are available to download at https://www.finngen.fi/en/access_results. GWAS summary statistics for the proteins of the INTERVAL cohort are available for download at: https://www.phpc.cam.ac.uk/ceu/proteins/. GWAS summary statistics for lipoprotein metabolomics parameters, from Kettunen et al. are available for download at: http://www.computationalmedicine.fi/data#NMR_GsWAS. The MetaXcan software is available to download at https://github.com/hakyimlab/MetaXcan. *TwoSampleMR* R package is available at https://mrcieu.github.io/TwoSampleMR/. *Coloc* R package is available at https://github.com/chr1swallace/coloc/. *Hyprcoloc* R package is available at https://github.com/jrs95/hyprcoloc/. *Gassocplot* R package is available at https://github.com/jrs95/gassocplot. *LocusCompareR* R package is available at https://github.com/boxiangliu/locuscomparer. PhenoSpD is available at https://github.com/MRCIEU/PhenoSpD. PhenomeXcan is available at http://apps.hakyimlab.org/phenomexcan/.
